# Comparative Genomic Analysis Determines the Functional Genes Related to Bile Salt Resistance in *Lactobacillus salivarius*

**DOI:** 10.3390/microorganisms9102038

**Published:** 2021-09-27

**Authors:** Qiqi Pan, Xudan Shen, Leilei Yu, Fengwei Tian, Jianxin Zhao, Hao Zhang, Wei Chen, Qixiao Zhai

**Affiliations:** 1 State Key Laboratory of Food Science and Technology, Jiangnan University, Wuxi 214122, China; Chelseapancn@outlook.com (Q.P.); ewingshen@163.com (X.S.); edyulei@126.com (L.Y.); fwtian@jiangnan.edu.cn (F.T.); jxzhao@jiangnan.edu.cn (J.Z.); zhanghao@jiangnan.edu.cn (H.Z.); chenwei66@jiangnan.edu.cn (W.C.); 2 School of Food Science and Technology, Jiangnan University, Wuxi 214122, China; 3 National Engineering Research Center for Functional Food, Jiangnan University, Wuxi 214122, China; 4 Wuxi Translational Medicine Research Center, Jiangsu Translational Medicine Research Institute Wuxi Branch, Wuxi 214122, China

**Keywords:** comparative genomics, *Lactobacillus salivarius*, bile salt tolerance

## Abstract

*Lactobacillus salivarius* has drawn attention because of its promising probiotic functions. Tolerance to the gastrointestinal tract condition is crucial for orally administrated probiotics to exert their functions. However, previous studies of *L. salivarius* have only focused on the bile salt resistance of particular strains, without uncovering the common molecular mechanisms of this species. Therefore, in this study, we expanded our research to 90 *L. salivarius* strains to explore their common functional genes for bile salt resistance. First, the survival rates of the 90 *L. salivarius* strains in 0.3% bile salt solutions were determined. Comparative genomics analysis was then performed to screen for the potential functional genes related to bile salt tolerance. Next, real-time polymerase chain reaction and gene knockout experiments were conducted to further verify the tolerance-related functional genes. The results indicated that the strain-dependent bile salt tolerance of *L. salivarius* was mainly associated with four peptidoglycan synthesis-related genes, seven phosphotransferase system-related genes, and one chaperone-encoding gene involved in the stress response. Among them, the GATase1-encoding gene showed the most significant association with bile salt tolerance. In addition, four genes related to DNA damage repair and substance transport were redundant in the strains with high bile salt tolerance. Besides, cluster analysis showed that bile salt hydrolases did not contribute to the bile salt tolerance of *L. salivarius*. In this study, we determined the global regulatory genes, including *LSL_1568*, *LSL_1716* and *LSL_1709*, for bile salt tolerance in *L. salivarius* and provided a potential method for the rapid screening of bile salt-tolerant *L. salivarius* strains, based on PCR amplification of functional genes.

## 1. Introduction

*Lactobacillus salivarius* was first isolated from children’s saliva in Maryland, USA [1]. Later studies have shown that it exists in various habitats, including the oral cavity, the gastrointestinal tract, and in the vagina of animals (including humans), as well as food materials [2,3,4].

In recent years, *L. salivarius* has drawn attention because of its promising probiotic functions, such as anti-aging, immunoregulation, and bacteriostasis [5]. A study indicated that *L. salivarius* FDB89, isolated from the feces of centenarians, extended the mean life span of *Caenorhabditis elegans* by up to 11.9% compared to that of the control group [6]. Furthermore, another *L. salivarius* strain, FXJCJ7-2, was shown to have significant anti-inflammatory effects on lipopolysaccharide (LPS)-treated murine macrophages and mice treated with LPS [7]. In addition, research focusing on combating *Staphylococcus aureus* infections found that *L. salivarius* KCTC3156 was a potential novel anti-staphylococcal agent that could be effective against *S. aureus* biofilms [8].

Tolerance to the gastrointestinal tract environment is a decisive factor for probiotics to exert physiological benefits in the gut. The adverse environment in the human intestine to probiotics is mainly caused by bile salts (average concentration of 0.3%) [9].

Bile salts can lead to high osmotic pressure and affect bacterial cells via protein denaturation, DNA damage, intracellular acidification, oxidative stress, and metabolic changes [10]. Bacteria have evolved a series of protective mechanisms to survive in the bile salt-rich environment of the digestive tract. Studies in this area have shown that bacterial bile salt resistance is strain-specific and that the mechanisms of bile salt resistance differ among strains.

Cell walls and envelopes are the first line of defense of bacteria against external agents. The cell surfaces of *L. plantarum* WCFS1 became rough and the whole cells began to atrophy under bile salt conditions [11]. Similar phenomena have been observed in *Propionibacterium freudenreichii* [12] and *L. acidophilus* [13]. The specific cell wall compositions of different strains contribute to their bile salt resistance, such as the external exopolysaccharide (EPS) and S-layers [14]. Bacterial efflux is the most direct mechanism of bacterial bile salt resistance. Bile salts accumulated in the cytoplasm can be extruded via this system to counteract bile toxicity. *L. acidophilus* strains, lacking the transporter genes belonging to the efflux system, exhibited a significant increase in sensitivity to bile [15]. In addition, bacteria possessing bile salt hydrolases (BSHs), which are enzymes belonging to the chologlycine hydrolase family, can survive in bile salt conditions by catalyzing bile salt deconjugation [16]. Due to the production of reactive oxygen species, bile salts impose oxidative stress on these bacterial strains. Bacteria activate chaperones to repair DNA/protein damage. Several studies have indicated that bacterial chaperones, including ClpP, Dps, GroEL, Hsp1, and Hsp3, were upregulated in bile salt environments [17]. Furthermore, a reorganization of the global metabolism to increase energy production can activate a response against the destructive action of bile salts at different concentrations, such as changing the cell wall architecture. A study of *L. johnsonii* PF01 reported that cell wall biosynthesis was improved in a bile salt environment [18].

With the rapid development in experimental techniques, a multi-omics method has been applied to bacterial bile salt resistance research, simplifying the exploration of strain-specific biomarkers. For example, a quantitative proteomic analysis of *L. fermentum* NCDC 400 identified 80 proteins responsive to the bile salt environment, including proteins that function in the stress response, DNA repair, peptidoglycan biosynthesis, and metabolism [19]. Another study integrating transcriptomic and proteomic analyses indicated that genes related to xylose utilization and bifid shunt were involved in the bile salt tolerance of *Bifidobacterium longum* BBMN68 [20]. In addition, research on *Bacillus coagulans* HS243 based on comparative genomic analysis showed two groups of bile-salt-tolerance-related genes, including a single-copy chologlycine hydrolase-encoding gene and four chaperone-related genes [21].

However, despite this expansion in the use of multi-omics techniques, research on *L. salivarius* in this field is still limited, and most of these studies only focused on a single strain. One study of *L. salivarius* LI01 revealed that its bile salt tolerance was related to cell envelope modification, regulatory systems, general stress response, and the central metabolic processes [22]. Another study on *L. salivarius* Ren reported similar results [23]. Notably, both studies mentioned above were based on proteomics and transcriptomics analyses, which are more expensive and more complicated to perform than genomics.

To determine the ability of strains’ bile salt resistance more efficiently, the survival rates of 90 *L. salivarius* strains in bile salts were tested. Then, the candidate functional genes for bile salt tolerance of *L. salivarius* were identified via comparative genomics analysis and verified via RT-PCR and gene knockout experiments. The result provided a potential method for the rapid screening of bile-salt-tolerant strains based on PCR amplification.

## 2. Materials and Methods

### 2.1. Isolation and Genome Sequencing of L. salivarius

The 90 *L. salivarius* strains used in this study were isolated from 500 stool samples of Chinese populations in 17 provinces. The stool samples were incubated in the modified medium under an anaerobic condition [7]. Each single strain was then picked and conducted a PCR reaction with a 16S rRNA common primer pair (Table S1) [24]. Sanger sequencing of the PCR products was performed by the Genewiz Laboratory (Suzhou, Jiangsu, China). The sequencing data were identified via Blast online (https://blast.ncbi.nlm.nih.gov/Blast.cgi (accessed on 10 November 2019)).

Strains classified into *L. salivarius* were cultured in the basic MRS broth for successive three generations at 37 °C. Bacterial genomic DNA was then extracted using a Rapid Bacterial Genomic DNA Isolation Kit (Sangon Biotech Co., Ltd., Shanghai, China) following the manufacturer’s instructions. Whole-genome sequencing was performed using an Illumina HiSeq platform (Illumina, Inc., San Diego, CA, USA) to generate 350-bp paired-end reads. High-quality sequences were assembled using SOAPdenovo2 (https://github.com/aquaskyline/SOAPdenovo2 (accessed on 28 December 2019)) [25]. The inner gaps in each scaffold were filled using GapCloser (https://github.com/BGI-Qingdao/TGS-GapCloser (accessed on 28 December 2019)) [26]. Genes were predicted using Genemark (http://exon.gatech.edu/GeneMark/license_download.cgi (accessed on 28 December 2019)) [27]. The genomic sequences of the 90 *L. salivarius* strains have been deposited in the NCBI database. The accession numbers of each strain are listed in Table S2.

This study was approved by the Ethics Committee in Jiangnan University, China (SYXK 2012-0002). All donors or their legal guardians provided written informed consent before sample collection. The collection of fecal samples caused no harm or discomfort to the participants. Detailed information about the 90 *L. salivarius* strains used in this study is listed in Table S2. The isolated strains were preserved at −80 °C in glycerol.

### 2.2. Determination of the Tolerance of L. salivarius Strains in Bile Salt Solutions

Bile salt solutions were prepared according to a published method, modified by changing the final concentration of bile salts to 0.3% [9].

Strains were grown overnight in basic MRS broth at 37 °C for 12 h. After subculturing twice, a 2% (*v*/*v*) inoculum of each strain was inoculated into 10 mL of fresh MRS broth and was grown for 12 h. The OD_600_ was measured, and the culture of each strain was diluted with MRS to the same absorbance. Cells in the diluted culture of each strain (1 mL) were collected via centrifugation (5000× *g*, 5 min) and washed twice with sterilized normal saline.

An aliquot (0.5 mL) of each washed cell suspension was used for the original viable count test. Cells in another 0.5 mL aliquot were collected via centrifugation (5000× *g*, 5 min) and mixed with a 0.3% bile salt solution (0.5 mL). The mixture was then incubated at 37 °C for 4 h. The total number of viable bacteria was determined after the tolerance assay. All experiments were conducted in triplicate. The survival rates of *L. salivarius* strains in bile salt solutions were calculated according to a previous method [28]. Briefly, the ratio of the total numbers of viable bacteria before and after incubation in the bile salt conditions was calculated for each strain.

### 2.3. Cluster Analysis of Three Subtypes of BSHs

Three subtypes of BSHs (BSHA, BSHB, BSHC), as described in a previous study [29], were identified in 90 *L. salivarius* strains using BLAST with e < 3.24e − 18 (https://github.com/mosuka/blast (accessed on 27 February 2020)) [30]. The phylogenetic trees of the three BSHs were constructed using MEGA-X (https://www.megasoftware.net/dload_win_gui (accessed on 28 February 2020)) [31] through the neighbor-joining method. Homologous sequences of BSHs from *L. johnsonii* PF01 were used as an outgroup to construct the phylogenetic trees (Table 1). The phylogenetic trees were visualized using Evolview v2 online software (www.evolgenius.info/evolview (accessed on 15 March 2020)).

### 2.4. Comparative Genomic Analysis

The top eight *L. salivarius* strains with high survival rates in the bile salt solutions (the tolerant group) and the bottom six strains with low survival rates (the non-tolerant group) were selected for comparative genomic analysis.

The core gene database of the tolerant group was built using OrthoMCL (https://orthomcl.org/orthomcl/ (accessed on 10 February 2020)) [32] with the default parameters. Next, the whole genome of each non-tolerant strain was compared with the tolerant core database using PGAP (https://github.com/ncbi/pgap (accessed on 15 February 2020)) [33], picking genes deleted in at least two strains via the result of orthologs clusters. Then, copy numbers of the deleting genes in each strain were analyzed using BLAST with its default parameters. Similarly, the redundant genes were determined via comparing the non-tolerant gene database with the tolerance strains’ genomes.

The protein sequences of all the genes picked above were predicted using Genemark (http://exon.gatech.edu/GeneMark/license_download.cgi (accessed on 9 February 2020)) [27]. Next, the functions of the protein sequences were annotated using the NR, CDD (https://ftp.ncbi.nih.gov/blast/db/ (accessed on 25 February 2020)), and COG (http://archive-dtd.ncbi.nlm.nih.gov/COG/ (accessed on 25 February 2020)) databases using BLAST with the default parameters. KEGG pathway annotations were conducted using DAVID 6.7 online (https://david.ncifcrf.gov/ (accessed on 25 February 2020)).

### 2.5. Quantitative RT-PCR

The strains with the highest and lowest survival rates were incubated in the bile salt solutions for 4 h after three subcultures, and a parallel culture grown in fresh MRS broth for the same duration was used as a control.

Cells in the culture of each strain (1 mL) were collected via centrifugation (8000× *g*, 10 min) in an enzyme-free tube and then mixed with 200 μL of lysozyme solution. The mixtures were incubated at 37 °C for 30 min to break the cell walls. Cells without cell walls were collected via centrifugation (12,000× *g*, 5 min, 4 °C). Total RNA from each strain was extracted using the TRIzol Plus RNA Purification Kit according to the manufacturer’s protocol (Invitrogen, Carlsbad, CA, USA). The RNA concentrations were measured using an RNA assay with a NanoDrop spectrophotometer (Thermo, Shanghai, China). cDNA was synthesized in a 20 μL reaction using a HiFiScript gDNA Removal cDNA Synthesis Kit (CoWin Biosciences, Wuxi, Jiangsu, China). qRT-PCR was performed in a 96-well plate following the TransStart Tip Green qPCR SuperMix kit (Tiangen Biotech Co., Ltd., Beijing, China) instruction. The thermocycling program was set as previously described [34], only changing the annealing temperature of the primers. The specific primers were designed using Primer3Plus online (http://www.primer3plus.com/ (accessed on 7 May. 2020)) and were synthesized by the Sangon Biotechnology Laboratory (Shanghai, China) (Table S1). The 16S rDNA was used as a reference gene [35]. All experiments were conducted in triplicate.

### 2.6. Gene Knockout of L. salivarius FWXBH36M1

A knockout experiment based on the Cre-lox system was performed according to a previously reported method, with minor modifications [36]. Briefly, 500-bp fragments of the upstream and downstream sequences of the target locus identified via RT-PCR were selected to construct gene-specific mutagenesis vectors (Figure S1a). The designed mutagenesis vectors were synthesized by the Exsyn-Biotechnology Laboratory (Wuxi, Jiangsu, China) and were preserved in *Escherichia coli* Top10 cells.

*E. coli* Top10 cells harboring the constructed plasmid were grown aerobically in Luria–Bertani (LB) broth at 37 °C. The plasmids were extracted using the GeneJET Plasmid Miniprep Kit (Thermo, Shanghai, China), following the manufacturer’s instructions. The plasmid concentrations were measured using a dsDNA assay with a NanoDrop spectrophotometer (Thermo, Shanghai, China).

Competent *L. salivarius* FWXBH36M1 cells were prepared according to a previously published method [37]. A 98-μL cell suspension and 2 μL of a plasmid DNA solution (containing approximately 2 μg plasmid) were electroporated in cuvettes with a 1-mm electroporation gap at a voltage of 2.2 kV, using an Electroporator 2510 system (Eppendorf, Shanghai, China). After transformation, 1 mL recovery buffer (MRS containing 1 mM MgCl_2_, 0.1 mM CaCl_2_, and 75.2 mM sorbitol) was added, and the cells were incubated for 4 h at 37 °C. Bacteria were grown on MRS plates containing 20 μg/mL chloramphenicol as a selective marker. The transformants were confirmed via PCR using vector-specific primers (Table S1) and agarose gel electrophoresis. Sanger sequencing of the amplified fragments was performed by the Genewiz Laboratory (Suzhou, Jiangsu, China).

To excise the chloramphenicol gene in the double-crossover mutants, the Cre expression vector with erythromycin-resistant gene was transformed into the mutants. The in-frame deletion mutants were selected as previously described with a minor change [36]. Briefly, after the transformation and recovery, bacteria were grown on MRS plates containing 15 μg/mL erythromycin as a selective marker. Then, the Em^r^ colonies were confirmed via PCR using erythromycin-specific primers (Table S1). The Cre expression vector was cured of Em^r^ colonies by growth without erythromycin selection pressure for 6 generations. Then, the mutants were confirmed via PCR using strain-specific primers (Table S1 and Figure S1b) and agarose gel electrophoresis. Sanger sequencing of the amplified fragments was performed by the Genewiz Laboratory (Suzhou, Jiangsu, China).

### 2.7. Statistical Analysis

Relative gene expression levels were calculated using the 2^−ΔΔCt^ method [38]. Differences between groups were analyzed via one-way ANOVA and the Kruskal–Wallis test using SPSS Statistics 25 (IBM, Armonk, NY, USA) and were visualized using GraphPad 8 software (GraphPad Software, San Diego, CA, USA). The heatmap package in R-4.0.2 was used to perform a hierarchical cluster analysis and visualize the different functional genes between the two groups.

## 3. Results

### 3.1. Different L. salivarius Strains Showed Diverse Survival Rates in Bile Salt Solutions

To compare the tolerance of different *L. salivarius* strains in bile salt solutions, their survival rates under the condition were analyzed. The results showed differences in intra-strain survival rates (Table S4). Among the 90 *L. salivarius* strains tested, the survival rates of eight strains exceeded 9.5%, where *L. salivarius* FWXBH36M1 showed the maximum survival rate (17.76%). The survival rates of six strains were ≤0.7%, and *L. salivarius* FYNDL5M1 exhibited the lowest survival rate (0.02%).

**Figure 1:**
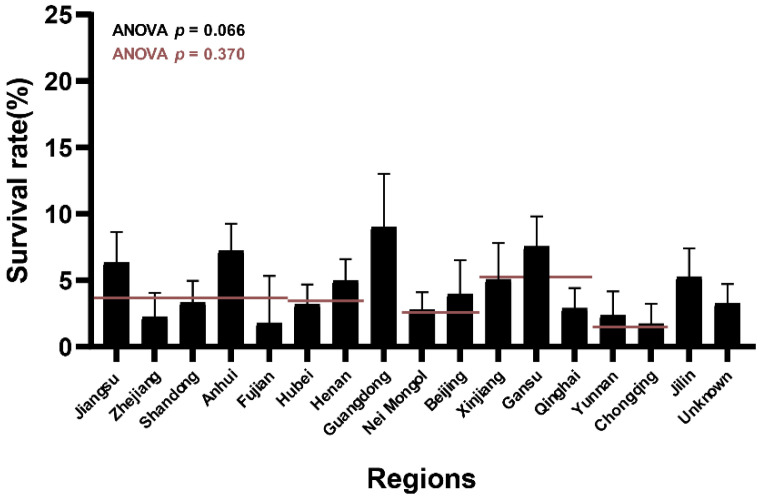
Average survival rates of *L. salivarius* strains isolated from different regions. The red line represents the average survival rates of the strains in different China part-groups. One-way ANOVA analysis was applied to compare the differences in survival rates in each region-group (*p*-values in black) and each China part-group (*p*-values in red).

Notably, this phenotypic variation was significant when the analysis was performed according to the isolated region of each strain (Figure 1, *p* < 0.10). Strains from the Guangdong, Gansu, Anhui, and Jiangsu provinces showed higher survival rates. In contrast, strains from the Fujian, Chongqing, Yunnan, and Zhejiang provinces showed lower survival rates. This tendency suggests that the tolerance of strains to bile salt solutions varied with their isolated regions. However, the area-dependent influence disappeared when the strains were classified according to the isolated parts of China (Figure 1, *p* > 0.10).

### 3.2. Strain Tolerance in Bile Salt Solutions Was Not Associated with BSH Variations

Previous studies have indicated that BSHs play a critical role in bacterial bile salt tolerance. To determine the contribution of different BSHs to the survival rates of *L. salivarius* strains in bile salt solutions, the different BSH types per strain were determined, and phylogenetic trees of the three BSH subtypes were constructed.

**Figure 2:**
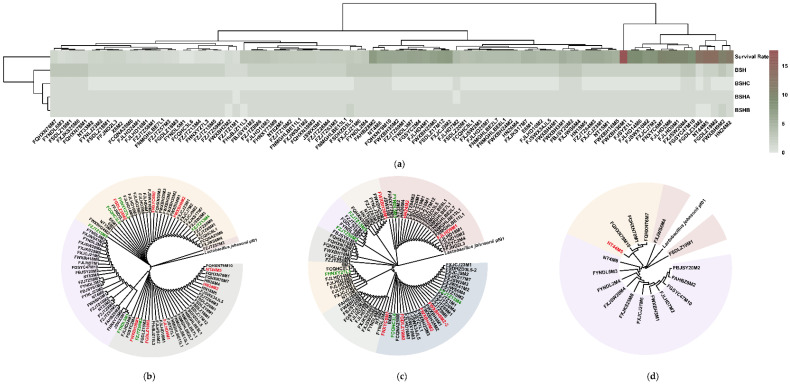
Number and phylogenetic trees of the three BSHs. (**a**) The number of three BSH types and the corresponding survival rate of each strain. The algorithm of hierarchical clustering was applied based on the Euclidean distance; (**b**–**d**) The phylogenetic trees of the strains’ BSHA, BSHB, and BSHC, respectively, using *L. johnsonii* PF01 as an outgroup. Strain names in red represent strains with survival rates higher than 9.5%; strain names in green represent strains with survival rates lower than 0.7%.

The results showed that 15 *L. salivarius* strains had all three BSH subtypes. The remaining 71 strains had only two types of BSHs (69 with BSHA and BSHB, one with BSHA and BSHC, and one with BSHB and BSHC). Among them, FWXBH9M2 and FSDHZD3L5 had double BSHB-encoding genes. The other four strains only had one type of BSH-encoding gene (three with BSHA only and one with BSHB only). However, the numbers and types of BSHs in each strain did not show region-dependent diversity (Figure 2a).

Hierarchical clustering indicated that changes in the number of BSHs genes followed a similar trend as the survival rates of the strains in bile salt solutions (Figure 2a). Then, each BSH subtype was further grouped according to its evolutionary distance. BSHA, BSHB, and BSHC had four, seven, and three groups, respectively. However, a further analysis combining the BSH groups based on their evolutionary distances and the survival rates of the strains in bile salt solutions did not reveal any similar patterns (Figure 2b–d and Table S4). Hence, the intra-strain diversity of bile salt tolerance in *L. salivarius* was not related to the variations in BSHs.

### 3.3. Comparative Genomic Analysis Determined the Potential L. salivarius Genes Responsible for Bile Salt Tolerance

To determine the potential mechanisms of how functional genes were involved in the bile salt tolerance of *L. salivarius*, a comparative genomic analysis of eight tolerant (survival rate ≥ 9.5%) and six non-tolerant (survival rate ≤ 0.7%) strains was performed.

**Figure 3:**
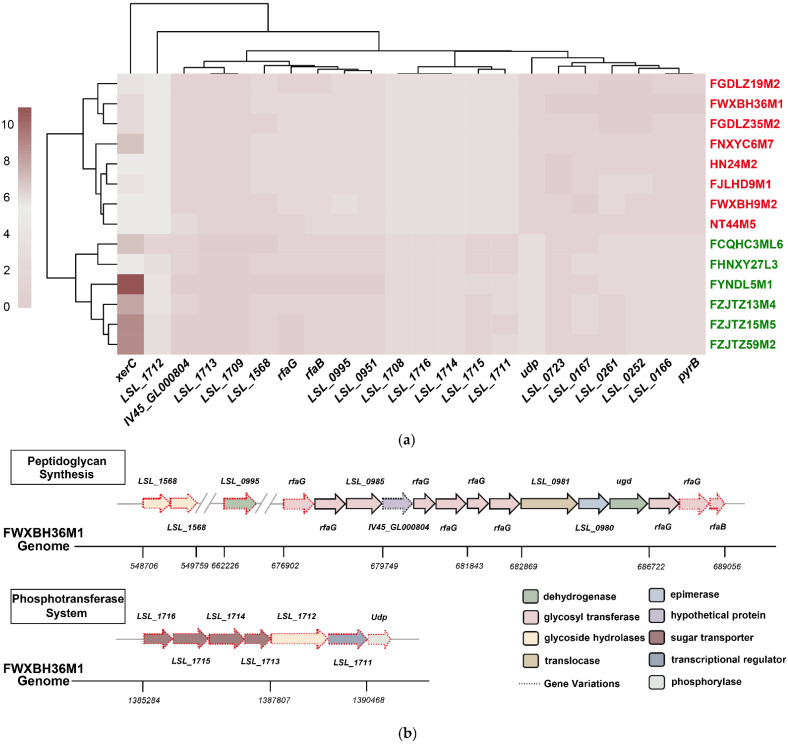
Comparative genomic analysis of 14 *L. salivarius* strains. (**a**) Differences in gene copy numbers in two groups of *L. salivarius* strains. Strain names in red represent strains with survival rates higher than 9.5%; strain names in green represent strains with survival rates lower than 0.7%. The algorithm of hierarchical clustering was applied based on the Euclidean distance; (**b**) Two gene clusters of functional genes in FWXBH36M1. Red frames mean genes are related to the mentioned pathway.

The results showed that there were 15 tolerant core genes deleting in at least two non-tolerant strains (Figure 3a). Among them, four were involved in carbohydrate transport, three encoded for different glycoside hydrolases, and six (two for each following function) were responsible for glycosyltransferase, transcription regulation, and hypothetical protein-encoding, respectively. The remaining two genes contributed to amidotransferase and phosphorylase encoding, respectively (Table 2). Further analysis via CD-BLAST revealed that the gene encoding amidotransferase (*LSL_1709*) was highly homologous with the Ydr533c protein-encoding genes in *Saccharomyces cerevisiae*, which are upregulated in response to various stress conditions [39].

Notably, further analysis of the gene functions showed that two functional clusters existed in the different genes, including four genes involved in peptidoglycan synthesis and seven genes in the phosphotransferase system (PTS) (Figure 3b). In the former cluster, three glycosyltransferase-related genes were involved in the acylation of N-acetylmuramic acid and N-acetylglucosamine, and one gene encoded β-N-acetylglucosaminidase, participating in the hydrolysis of chitin (Table 2). KEGG analysis identified that the latter cluster was enriched in mannose- and N-acetylglucosamine-specific PTS pathways. In particular, two genes (*LSL_1716* and *LSL_1715*) encoding the PTS system transporter subunits IIB and IIC, respectively, were involved in mannose transmembrane transport. Two genes (*LSL_1714* and *LSL_1713*), which encode for the PTS system transporter subunits IIABC and IIBC, respectively, are involved in N-acetylglucosamine transmembrane transportation (Table 2, Figure S3).

### 3.4. Possible Redundant L. salivarius Genes for Bile Salt Tolerance Were Identified via Comparative Genomic Analysis

Next, the whole genomes of the tolerant strains were compared with the core genes of the non-tolerant group using PGAP to determine the redundant genes under bile salt solutions. The results showed seven different genes between the two groups, including one TetR/AcrR family transcriptional regulator-encoding gene, one site-specific integrase-encoding gene, one aspartate carbamoyltransferase-encoding gene, and four genes that encode hypothetical proteins (Table 2). The copy numbers of all seven genes were reduced in the bile salt-tolerant strains (Figure 3a).

After combining the annotations of the upstream and downstream genes of the unknown genes, it could be inferred that *LSL_0261*, *LSL_0166*, and *LSL_0167* were related to substance metabolism and transportation (Figure S4). In addition, CD-BLAST showed that the *LSL_0252*-encoding protein belongs to the HTH_17 superfamily, involved in DNA recombination.

### 3.5. qRT-PCR Showed the Transcriptional Changes of the Possible Functional and Redundant Genes Related to Tolerance in Bile Salt Solutions

qRT-PCR was conducted on bile salt-tolerant (FWXBH36M1 and FJLHD9M1) and non-tolerant strains (FYNDL5M1 and FCQHC3ML6) to determine the transcriptional changes of the functional and redundant genes identified above upon culture in bile salt solutions. According to their genome coordinates and functions, the tolerance-related genes could be classified into five groups (Table S3), and one target gene from each group was selected for qRT-PCR. qRT-PCR for the redundant genes was performed after excluding genes with high copy number changes in both groups (*pyrB* and *xerC*) (Figure 3a).

**Figure 4:**
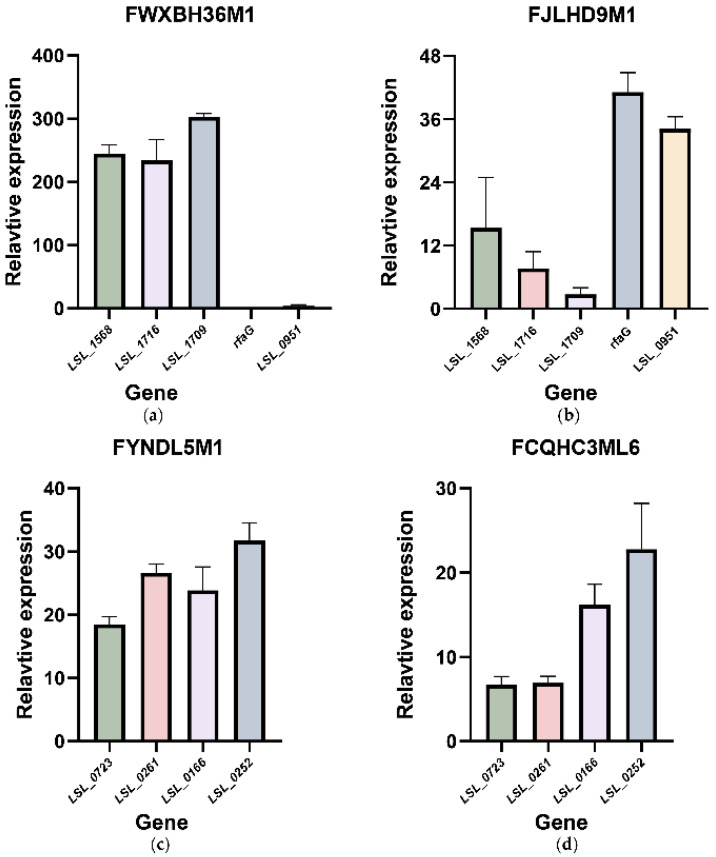
qRT-PCR of the bile salt resistance-related genes in *L. salivarius*. (**a**,**b**) Relative expression of the functional genes conferring bile salt resistance in *L. salivarius* strains FWXBH36M1 and FJLHD9M1; (**c**,**d**) Relative expression of the redundant genes conferring bile salt resistance in *L. salivarius* strains FYNDL5M1 and FCQHC3ML6.

For the tolerance-related genes, the results showed that the relative expression level of *LSL_1568*, *LSL_1716*, *LSL_1709*, and *LSL_0951* were increased in both strains when strains were grown in bile salt solutions (Figure 4a,b). However, the relative expression level of *rfaG* showed a slight decrease in FWXBH36M1 (0.18 on average); in FJLHD9M1, it showed a maximum increase (41.12 on average).

The four genes with increased relative expression levels were changed to different extents in the two tested strains. In FWXBH36M1, the relative expression of *LSL_1568*, *LSL_1716*, and *LSL_1709* showed remarkable changes compared with that of *LSL_0951*. There was an opposite trend in the relative expression level of these functional genes in FJLHD9M1, where *LSL_0951* demonstrated a higher expression level than others. The differences in the fold-changes of the functional genes upon growth in a bile salt system may explain the intra-strain diversity in the bile salt tolerance of *L. salivarius*.

In addition, an inconsistency in the relative expression of the redundant genes was found in strains FYNDL5M1 and FCQHC3ML6, despite the noticeable growth of all four genes (Figure 4c,d). This inconsistency may have resulted in the fluctuations in the survival rates in the non-tolerant strains.

### 3.6. Knockout of the Functional Genes in L. salivarius FWXBH36M1 Verified Their Contributions to the Bile Salt Tolerance

To prove that the functional genes obtained above influenced the tolerance of *L. salivarius* to bile salt solutions, in-frame deletions of the top three most highly relatively expressed genes in FWXBH36M1 were performed. Confirmation of positive transformants via DNA electrophoresis is shown in Figure S5.

**Figure 5:**
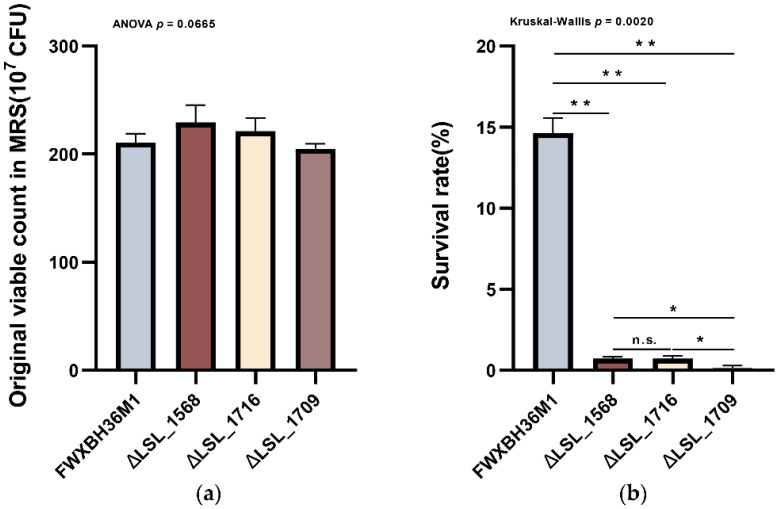
Knockout of the functional genes in *L. salivarius* strain FWXBH36M1. (**a**) Growth profiles of three in-frame deletion mutants and the wild-type strain in MRS medium. One-way ANOVA was applied for the group comparison; (**b**) Survival rates of three in-frame deletion mutants and the wild-type strain in 0.3% bile salt solution. Kruskal–Wallis test and Tamhane T2 *post hoc* analysis were applied for the group comparison. Symbols indicate significance (** *p* < 0.01, * *p* < 0.05).

Compared with that of the wild-type strain, the growth profile of the three mutant strains in MRS did not show any significant differences (Figure 5a, *p* > 0.05), indicating that gene knockout did not affect the growth of the strains under normal conditions.

However, the survival rates of the three mutant strains were significantly lower than that of the wild-type strain under bile salt conditions (Figure 5b, *p* < 0.05). Additionally, the survival rates of the three mutant strains showed a consistent trend with the relative expression of the corresponding genes. Specifically, the knockout of the most highly expressed gene in FWXBH36M1 also led to the most significant drop-in survival rate.

Therefore, the functional genes obtained through the comparative genomic analysis were related to the survival rates of *L. salivarius* strains in bile salt solutions.

## 4. Discussion

In this study, we identified the potential functional genes that confer bile salt tolerance in *L. salivarius* via comparative genomic analysis and verified the results via qRT-PCR and gene knockout experiments.

Ninety *L. salivarius* strains showed intra-strain differences in survival rates in bile salt solutions, indicating that intrinsic bile salt tolerance is strain-specific. Similar results have been reported in previous studies on *Bifidobacterium* [40] and *L. acidophilus* strains [41]. Considering the open pan-genome of *L. salivarius* [42], phenotypic differences might have resulted from gene variations. The region-level analysis demonstrated that the phenotypic differences were area-dependent, which might be attributed to the niche-specific adaptive evolution of *Lactobacillus* [43]. Notably, as the grouping area expanded further, this influence disappeared. Similar results have been reported for *L. plantarum* [44], thus suggesting that the grouping level of strain habitats greatly influences the results of genetic evolution analysis.

Further analysis of BSH variations did not reveal any associations between the BSHs and the bile salt resistance of *L. salivarius* strains, thus indicating that intra-strain phenotypic differences in bile salt tolerance were induced by mechanisms other than the presence of the BSHs. Similar results have been reported in previous studies on *L. johnsonii* [18] and *L. salivarius* [6], indicating that bacterial bile salt resistance is a multifactorial phenomenon [10].

Comparative genomic analysis based on the whole bacterial genome showed that the bile salt resistance of *L. salivarius* strains was associated with the peptidoglycan synthesis process. Peptidoglycan is the main component of the cell wall [45]. The cell wall is the first line of defense for microorganisms against the external environment [10]. Therefore, strains with a higher abundance of genes related to cell wall synthesis potentially have stronger survival abilities in bile salt solutions. Several studies on *L. fermentum* [19], *L. johnsonii* [18], and *L. plantarum* [11] found similar results regarding the transcription level of these genes under bile salt conditions.

Comparative genomic analysis and KEGG enrichment analysis indicated that bacterial PTS also played an important role in the bile salt resistance of *L. salivarius* strains. PTS is a complex kinase system that catalyzes sugar transport and phosphorylation [46]. It is composed of the enzyme I, enzyme II, and HPr (heat-stable). Enzyme II is further composed of the A, B, and C subunits. Our results showed that the bile salt-tolerant strains had a mannose-specific translocator-encoding gene. Notably, mannose is inefficient in terms of energy supply, so most bacteria do not use this sugar for energy supplementation [47]. In a stressful environment, however, bacteria tend to extensively use various carbon sources in the environment to meet their survival needs [20]. Therefore, strains enriched with energy intake genes have a potential survival advantage.

Additionally, the relative expression of *LSL_1709* in the strain with maximum stress tolerance increased dramatically in the bile salt condition, and the survival rate of *LSL_1709*-mutant strain significantly lowered in the bile salt solutions. All of these indicated this gene has a strong association with the strains’ bile salt-tolerant ability. Considering the chaperone activity of *LSL_1709*, its encoding product may involve the strains’ bile salt tolerance by inhibiting the misfolding and denaturation of protein caused by the stress condition [48].

Analysis of the strains’ redundant genes related to bile salt tolerance showed that four potential redundant genes were related to the repair of DNA damage, substance transportation and transcription regulation. All of these can involve repairing bacterial damage caused by bile salts [49,50]. This may explain the up-regulated expression of the four genes in the bile salt condition. However, for the strains with high bile salt tolerance, the four genes exhibited significant variations, which implies that these genes are involved in the downstream pathways of bile resistance regulation. Also, considering the redundant metabolism-related genes were involved in the amino acid usage, which is a priority energy utilization strategy. Therefore, all these genes were not necessary for tolerant strains to keep these genes.

Interestingly, the relative expressions of the possible functional and redundant genes varied from strain to strain, indicating that even the same gene has different responses to bile salt conditions in different strain’s genomic backgrounds. This phenomenon can also be observed in *S. cerevisiae* [51] and *Bacillus mycoides* [52], which may be the reason for the huge differences in the phenotypes of strains. Therefore, in future functional genome studies, we should fully consider the contribution weight of different functional genes to the specific phenotype in different bacterial genome backgrounds and develop an appropriate phenotypic prediction scheme based on this, which will effectively improve the accuracy of the results.

However, it should be noticed that this research was based on in vitro experiments only, lacking the corresponding in vivo confirmation. Previous studies have shown that the maximum concentrations of bile salts tolerated by probiotics differ in vivo and in vitro. Besides, the types of bile salts used and the bile conditions in the in vitro experiments were different from those in the human intestine [53]. Therefore, it is necessary to assess the strain’s tolerant ability in the host condition where they are used to precisely uncover the host-specific tolerant mechanisms of probiotic strains.

Overall, in this study, we applied comparative genomic analyses to determine the mechanism of how the gene variations involved in the bile salt tolerance of *L. salivarius*. The results indicated that the bile salt resistance of *L. salivarius* strains was mainly related to the chaperones, PTS and peptidoglycan synthesis. Our work also provided a potential method for rapidly screening the bile-salt-tolerant strains of *L. salivarius* based on PCR of the functional genes.

## Figures and Tables

**Table 1:** Information on the three BSH subtypes from *L. johnsonii* PF01.

Subtypes	Function	Accession No.
BSHA	Taurine-conjugated bile salt hydrolase	EGP12224
BSHB	Taurine-conjugated bile salt hydrolase	EGP13287
BSHC	Glycosyl-conjugated bile salt hydrolase	EGP12391

**Table 2:** Functions of the different genes identified between the two groups of *L. salivarius* strains.

Group	Gene	Function
Variable Genes	*LSL_1568*	beta-N-acetylhexosaminidase
	*LSL_0995*	UDP-D-quinovosamine 4-dehydrogenase
	*rfaG*	glycosyl transferase
	*IV45_GL000804*	hypothetical protein
	*rfaB*	glycosyl transferase
	*LSL_0951*	hypothetical protein
	*LSL_1716*	PTS mannose transporter subunit IIB
	*LSL_1715*	PTS sugar transporter subunit IIC
	*LSL_1714*	PTS N-acetylglucosamine transporter subunit IIABC
	*LSL_1713*	PTS N-acetylglucosamine transporter subunit IIBC
	*LSL_1712*	alpha-glucosidase
	*LSL_1711*	LacI family transcriptional regulator
	*LSL_1709*	type 1 glutamine amido transferase
	*LSL_1708*	ArsR family transcriptional regulator
	*Udp*	phosphorylase
Redundant Genes	*LSL_0723*	transcriptional regulator
	*LSL_0261*	hypothetical protein
	*LSL_0166*	hypothetical protein
	*LSL_0167*	hypothetical protein
	*pyrB*	aspartate carbamoyltransferase catalytic subunit
	*xerC*	site-specific integrase
	*LSL_0252*	hypothetical protein

## Data Availability

All the strains sequences used in the article have been deposited in the NCBI database. Accessions numbers by strains are listed in Table S2. The 22 short reads picked from two strains can be found in the bioproject PRJNA658852. All the codes used in this research can be found at Github (https://github.com/Chelseapan/CGA (accessed on 16 September 2021)).

## Supplementary Materials

The following are available online at https://www.mdpi.com/article/10.3390/microorganisms9102038/s1, Table S1: Primer sequences; Table S2: Detailed information of 90 *Lactobacillus salivarius* strains; Table S3: Five groups of functional genes; Table S4: Survival rates of 90 *L. salivarius* strains in 0.3% bile salt solutions; Figure S1: The gene knockout strategy; Figure S2: The isolated regions of 90 *L**. salivarius* strains; Figure S3: The specific PTS pathway in two group of strains identified by KEGG; Figure S4: The upstream and downstream genes of the unknown redundant genes and their functions; Figure S5: The DNA electrophoresis of three mutant strains.
