# Supplementary material for: Comparative Genomic Analysis Determines the Functional Genes Related to Bile Salt Resistance in Lactobacillus salivarius
Source: Microorganisms. 2021 Sep 27;9(10):2038. doi: 10.3390/microorganisms9102038 (PMC8539994; doi:10.3390/microorganisms9102038)
Supplement: Supplementary file 1 [file microorganisms-09-02038-s001.zip › microorganisms-1367084-supplementary.pdf]

Table S1. Primer sequences.

Primer	Sequence (5'-3')
**Strain identification**	
27F	AGAGTTTGATCMTGGCTCAG
1492R	CGGTTACCTTGTTACGACTT
**Bile salt tolerance test**	
LSL_1568-F	GAAAGTTTCCTTCCCCACAAGAAG
LSL_1568-R	CAACTGGTGCAAAGTCCCAATT
rfaG-F	GCGGCTCAAAAAGATGAACGAT
rfaG-R	CCAGAAGTCATTGTCCAGGGAAT
LSL_0951-F	GGATGCTCCTAAAGAGGTTAAAGC
LSL_0951-R	GAAACTTGTCCATCCCCCATTTC
LSL_1716-F	CTGCAGGAACAGGAATGTCAATC
LSL_1716-R	GGTATTTTCACACCACCCCTGAT
LSL_1709-F	GACAGGTTTGTGGTTAGCTGAAG
LSL_1709-R	CGAGGATCTAGAGGAACTTCTCC
LSL_0723-F	AGTGGCACCCTAGAAGAATTGAG
LSL_0723-R	GCTTGATTTTGGTCTATCTGCGAC
LSL_0261-F	GCATCAGCAGCAGATGTTAAAG
LSL_0261-R	CCATCAGCTTCAAAGATTGCTCC
LSL_0166-F	GTCTCAGGGATAGCCTTGTTGAT
LSL_0166-R	CCGCATACTTATATCCTCCTGCA
LSL_0252-F	CATAACAGAAGCAGCTAAGCACTG
LSL_0252-R	CCCCACCAGCTGTTAGTTCAATA
Uni33F	TCCTACGGGAGGCAGCAGT
Uni797R	GGACTACCAGGGTATCTATCCTGTT
**Gene knockout test**	
up-test-R	GTTTTTTTCTAGTCCAAGCTCACA
down-test-F	GCCGACTGTACTTTCGGATCCT
LSL_1568-test-F	GCATCGGATGGATTTGTAATCC
LSL_1568-test-R	CCTGCATCAGTTAAAATTATCGG
LSL_1709-test-F	GGACAGTTAATACTGACAGTGA
LSL_1709-test-R	CGCGGACACCACC
LSL_1716-test-F	CCCACTAGGATTATTTACGGTT
LSL_1716-test-R	CACATCTTCCATTGTTCCG
Ery-F	.CGATACCGTTTACGAAATTGG
Ery-R	CTTGCTCATAAGTAACGGTAC

Table S2. Detailed information of 90 *Lactobacillus salivarius* strains.

Strain	Region (City / Province)	Origin	Accession No.
NT15M1	Nantong, Jiangsu	Human feces	GCA_009863885.1
NT33M2	Nantong, Jiangsu	Human feces	GCA_009863585.1

Strain	Region (City / Province)	Origin	Accession No.
NT4M8	Nantong, Jiangsu	Human feces	GCA_009863835.1
NT62M5	Nantong, Jiangsu	Human feces	GCA_009863815.1
NT44M5	Nantong, Jiangsu	Human feces	SRR12559554
FJSWX10M2	Wuxi, Jiangsu	Human feces	GCA_009863685.1
FJSWX34JL5	Wuxi, Jiangsu	Human feces	GCA_009863705.1
FWXBH24M2	Wuxi, Jiangsu	Human feces	GCA_009863805.1
FWXBH25M3	Wuxi, Jiangsu	Human feces	GCA_009863785.1
FWXBH2M2	Wuxi, Jiangsu	Human feces	GCA_009863715.1
FWXBH35M2	Wuxi, Jiangsu	Human feces	GCA_009869955.1
FWXBH36M1	Wuxi, Jiangsu	Human feces	SRR12559766
FWXBH3M1	Wuxi, Jiangsu	Human feces	GCA_009863465.1
FWXBH4M1	Wuxi, Jiangsu	Human feces	GCA_009863735.1
FWXBH9M2	Wuxi, Jiangsu	Human feces	SRR12559767
JSWX5M1	Wuxi, Jiangsu	Human feces	GCA_009863695.1
FWXBH18M5	Wuxi, Jiangsu	Human feces	GCA_009863625.1
FJSYZ1L1	Yangzhou, Jiangsu	Human feces	SRR12559777
FZJTZ10M2	Taizhou, Zhejiang	Human feces	GCA_009863635.1
FZJTZ13M4	Taizhou, Zhejiang	Human feces	GCA_009863605.1
FZJTZ15M5	Taizhou, Zhejiang	Human feces	SRR12559761
FZJTZ1M1	Taizhou, Zhejiang	Human feces	GCA_009863555.1
FZJTZ28M4	Taizhou, Zhejiang	Human feces	GCA_009863535.1
FZJTZ57M3	Taizhou, Zhejiang	Human feces	SRR12559554
FZJTZ58M1	Taizhou, Zhejiang	Human feces	SRR12559558
FZJTZ59M2	Taizhou, Zhejiang	Human feces	SRR12559557
FZJTZ63M3	Taizhou, Zhejiang	Human feces	SRR12559556
FZJTZ64M3	Taizhou, Zhejiang	Human feces	GCA_009865735.1
FZJTZ69M1	Taizhou, Zhejiang	Human feces	GCA_009870175.1
FZJTZ9M6	Taizhou, Zhejiang	Human feces	GCA_009865705.1
FSDHZD3L5	Hezhe, Shandong	Human feces	GCA_009863485.1
FSDLZ17M12	Laizhou, Shandong	Human feces	GCA_009870365.1
FSDLZ18M1	Laizhou, Shandong	Human feces	GCA_009865815.1
FSDLZ19M1	Laizhou, Shandong	Human feces	SRR12560180
FSDLZ20M1	Laizhou, Shandong	Human feces	SRR12559768
FAHBZ8M2	Haozhou, Anhui	Human feces	GCA_009863415.1
FFJND2L3	Ningde, Fujian	Human feces	SRR12559781
FHuBJZ11L3	Jingzhou, Hubei	Human feces	GCA_009863365.1
FHNXY27L3	Xinyang, Henan	Human feces	GCA_009863405.1
FHNXY73M9	Xinyang, Henan	Human feces	GCA_009869975.1
HN18M5	Unknown, Henan	Human feces	SRR12559555
HN24M2	Unknown, Henan	Human feces	GCA_009870225.1
HN26M4	Unknown, Henan	Human feces	GCA_009866085.1
FGDLZ18M3	Leizhou, Guangdong	Human feces	GCA_009870335.1

Strain	Region (City / Province)	Origin	Accession No.
FGDLZ19M2	Leizhou, Guangdong	Human feces	SRR12559779
FGDLZ35M2	Xining, Qinghai	Human feces	GCA_009866275.1
FNMGHLBE11L1	Hulunbeier, Nei Mongol	Human feces	SRR12559776
FNMGHLBE13L1	Hulunbeier, Nei Mongol	Human feces	GCA_009870285.1
FNMGHLBE2L7	Hulunbeier, Nei Mongol	Human feces	GCA_009870295.1
FNMGHLBE7L1	Hulunbeier, Nei Mongol	Human feces	GCA_009866265.1
FNMGHLBE8L1	Hulunbeier, Nei Mongol	Human feces	GCA_009863295.1
FBJSY20M2	Shunyi, Beijing	Human feces	GCA_009866235.1
FBJSY61M2	Shunyi, Beijing	Human feces	SRR12559783
FXJCJ21M6	Changji, Xinjiang	Human feces	SRR12559765
FXJCJ23M1	Changji, Xinjiang	Human feces	GCA_009866205.1
FXJCJ7M2	Changji, Xinjiang	Human feces	GCA_009865985.1
FXJCJ9M2	Changji, Xinjiang	Human feces	GCA_009870255.1
FXJKS17M7	Kashi, Xinjiang	Human feces	GCA_009866185.1
FXJKS25M8	Kashi, Xinjiang	Human feces	GCA_009866125.1
FXJSW20M4	Shawan, Xinjiang	Human feces	SRR12559764
FXJSW2M3	Shawan, Xinjiang	Human feces	GCA_009866115.1
FXJWS41M2	Wusu, Xinjiang	Human feces	GCA_009866135.1
FXJWS6M4	Wusu, Xinjiang	Human feces	GCA_009866105.1
FGSYC2M4	Yongchang, Gansu	Human feces	SRR12559778
FGSYC47M10	Yongchang, Gansu	Human feces	GCA_009866015.1
FQHXN39M3	Xining, Qinghai	Human feces	SRR12559775
FQHXN76M7	Xining, Qinghai	Human feces	SRR12559773
FQHXN78M1	Xining, Qinghai	Human feces	SRR12559772
FQHXN79M10	Xining, Qinghai	Human feces	SRR12559771
FNXYC6M7	Yinchuan, Ningxia	Human feces	GCA_009865995.1
FYNDL2M4	Dali, Yunnan	Human feces	GCA_009865965.1
FYNDL3M7	Dali, Yunnan	Human feces	SRR12559762
FYNDL5M1	Dali, Yunnan	Human feces	GCA_009869985.1
FYNDL6M3	Dali, Yunnan	Human feces	GCA_009866035.1
FYNLJ23M2	Lijiang Yunnan	Human feces	GCA_009865755.1
FCQHC3L6	Hechuan, Chongqing	Human feces	SRR12559782
FCQHC8L1	Hechuan, Chongqing	Human feces	GCA_009865895.1
FCQNA25M6	Nanan, Chongqing	Human feces	GCA_009865885.1
FJLHD10M2	Huadian, Jilin	Human feces	GCA_009865865.1
FJLHD14M6	Huadian, Jilin	Human feces	GCA_009863905.1
FJLHD16M3	Huadian, Jilin	Human feces	GCA_009865785.1
FJLHD18M1	Huadian, Jilin	Human feces	GCA_009863515.1
FJLHD24M1	Huadian, Jilin	Human feces	GCA_009863595.1
FJLHD25M7	Huadian, Jilin	Human feces	GCA_009863435.1
FJLHD2M8	Huadian, Jilin	Human feces	GCA_009870215.1
FJLHD4M1	Huadian, Jilin	Human feces	GCA_009866025.1

Strain	Region (City / Province)	Origin	Accession No.
FJLHD7M2	Huadian, Jilin	Human feces	GCA_009865905.1
FJLHD9M1	Huadian, Jilin	Human feces	GCA_009865925.1
6M2	Unknown	Human feces	GCA_009870355.1
E6M1	Unknown	Human feces	GCA_009863495.1

Table S3. Five groups of functional genes.

Group	Gene
PTS related genes	*LSL_1716*
	LSL_1715
	LSL_1714
	LSL_1713
	LSL_1712
	LSL_1711
	Udp
hydrolases	*LSL_1568*
	LSL_0995
	LSL_1712
glycosyl transferase	*rfaG*
	rfaB
glutamine amido transferase	*LSL_1709*
hypothetical protein	*IV45_GL000804*
	LSL_0951

*NB: Red represents genes tested by qRT-PCR.

Table S4. Survival rates of 90 *L. salivarius* strains in 0.3% bile salt solutions.

Strain	Survival rates (%) (Mean ± SD)	Strain	Survival rates (%) (Mean ± SD)
NT15M1	5.49 ± 2.62	FGDLZ18M3	0.80 ±3.50
NT33M2	1.70 ± 1.36	FGDLZ19M2	13.49 ± 4.50
NT4M8	6.19 ±1.19	FGDLZ35M2	12.71 ± 3.97
NT62M5	2.64 ± 0.71	6M2	1.80 ± 0.85
NT44M5	13.50 ± 3.78	E6M1	4.68 ± 1.91
FJSWX10M2	7.78 ± 1.61	FNMGHLBE11L1	2.55 ±1.02
FJSWX34JL5	4.43 ± 2.02	FNMGHLBE13L1	2.39 ±1.51
FWXBH24M2	4.02 ± 2.85	FNMGHLBE2L7	3.88 ± 1.92
FWXBH25M3	6.79 ± 2.02	FNMGHLBE7L1	1.21 ± 0.80
FWXBH2M2	1.17 ± 0.90	FNMGHLBE8L1	3.89 ± 1.21
FWXBH35M2	6.22 ± 2.48	FBJSY20M2	4.84 ± 0.71
FWXBH36M1	17.76 ± 2.66	FBJSY61M2	3.10 ± 3.54
FWXBH3M1	5.38 ±1.41	FJLHD7M2	4.26 ± 1.61
FWXBH4M1	4.39 ± 2.12	FJLHD9M1	9.72 ± 1.63

Strain	Survival rates (%) (Mean ± SD)	Strain	Survival rates (%) (Mean ± SD)
FWXBH9M2	10.76 ± 3.53	FXJCJ21M6	2.40 ± 2.07
JSWX5M1	2.30 ± 2.83	FXJCJ23M1	5.88 ± 4.09
FWXBH18M5	5.17 ± 2.12	FXJCJ7M2	7.94 ± 1.19
FJSYZ1L1	8.61 ± 2.02	FXJCJ9M2	7.32 ± 4.07
FZJTZ10M2	2.65 ± 1.75	FXJKS17M7	4.75 ± 3.78
FZJTZ13M4	0.57 ± 0.50	FXJKS25M8	1.24 ± 0.51
FZJTZ15M5	0.46 ± 0.74	FXJSW20M4	9.66 ± 3.68
FZJTZ1M1	0.79 ± 0.85	FXJSW2M3	4.12 ± 1.78
FZJTZ28M4	2.29 ± 1.95	FXJWS41M2	2.50 ± 1.85
FZJTZ57M3	1.01 ± 1.50	FXJWS6M4	4.81 ± 1.36
FZJTZ58M1	1.26 ± 1.50	FGSYC2M4	6.42 ± 1.41
FZJTZ59M2	0.39 ± 0.50	FGSYC47M10	8.66 ± 2.90
FZJTZ63M3	2.43 ± 2.12	FQHXN39M3	2.5 ± 1.78
FZJTZ64M3	5.83 ± 3.26	FQHXN76M7	1.56 ± 1.11
FZJTZ69M1	6.17 ± 2.12	FQHXN78M1	1.24 ± 1.78
FZJTZ9M6	3.24 ±1.61	FQHXN79M10	6.25 ± 1.36
FSDHZD3L5	2.69 ±1.54	FNXYC6M7	10.08 ± 1.41
FSDLZ17M12	7.16 ± 2.50	FYNDL2M4	2.78 ± 2.36
FSDLZ18M1	1.93 ± 1.01	FYNDL3M7	6.52 ± 2.66
FSDLZ19M1	1.18 ± 1.21	FYNDL5M1	0.02 ± 0.51
FSDLZ20M1	3.75 ± 1.61	FYNDL6M3	0.88 ± 0.97
FAHBZ8M2	7.23 ± 2.02	FYNLJ23M2	1.63 ± 1.50
FFJND2L3	1.78 ± 3.54	FCQHC3L6	0.04 ± 0.54
FHuBJZ11L3	3.19 ±1.50	FCQHC8L1	3.78 ± 2.49
FHNXY27L3	0.62 ±1.75	FCQNA25M6	1.35 ± 0.51
FHNXY73M9	2.78 ± 1.50	FJLHD10M2	4.3 ± 2.95
HN18M5	5.73 ±1.24	FJLHD14M6	8.73 ± 0.71
HN24M2	11.84 ±2.02	FJLHD16M3	3.25 ± 1.61
HN26M4	3.96 ±1.42	FJLHD18M1	1.15 ± 3.50
FJLHD2M8	9.66 ± 0.71	FJLHD24M1	1.35 ± 1.02
FJLHD4M1	6.32 ± 2.78	FJLHD25M7	3.91 ± 2.01

*NB: Strain names in red represent strains with survival rates higher than 9.5%; strain names in green represent strains with survival rates lower than 0.7%.

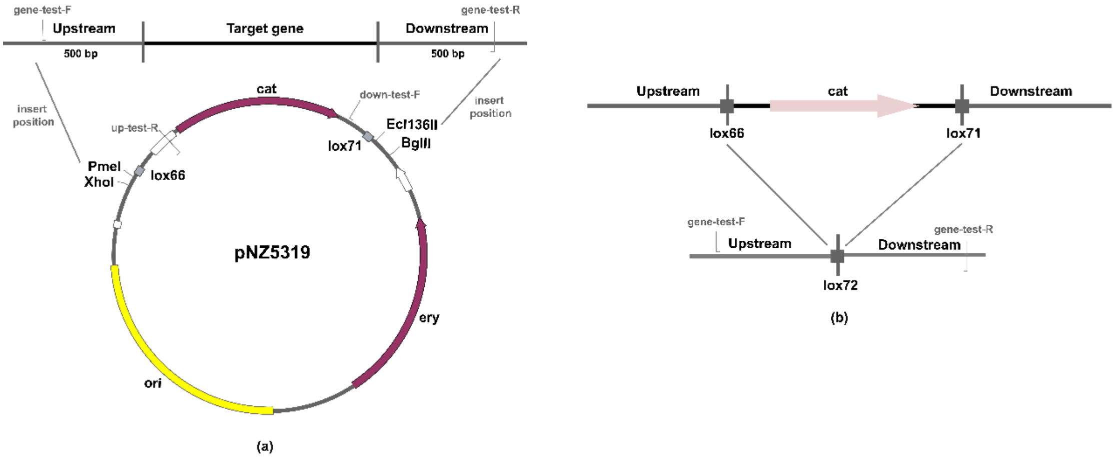

Figure S1. The gene knockout strategy. (a) The construction of gene-specific vector. Target gene represents *LSL_1568*, *LSL_1716* and *LSL_1709*. Gene-test-F and gene-test-R represent the test primers for the upstream and downstream sequences respectively in the transformants. Up-test-R and down-test-F represent the test primers for the plasmid sequence in the transformants. (b) Cre-mediated mutant locus resolution. Gene-test-F and gene-test-R represent the same primers in (a).

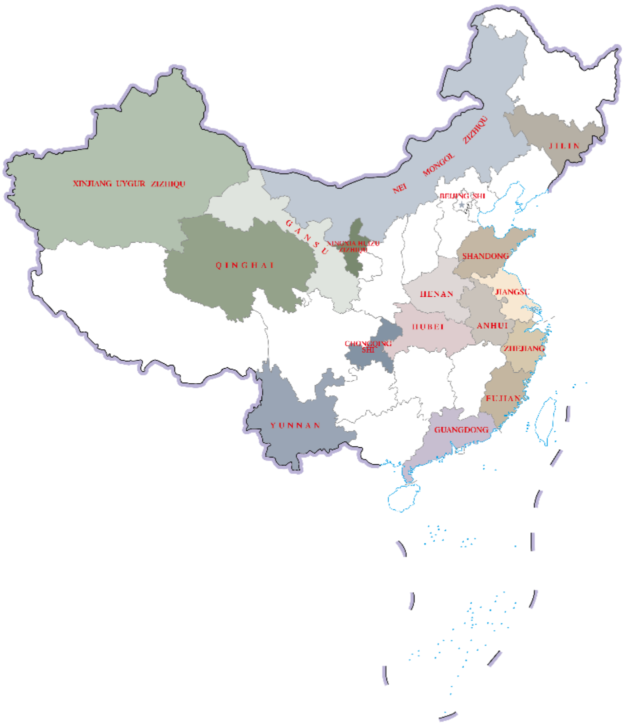

Figure S2. The isolated regions of 90 *Lactobacillus salivarius* strains.

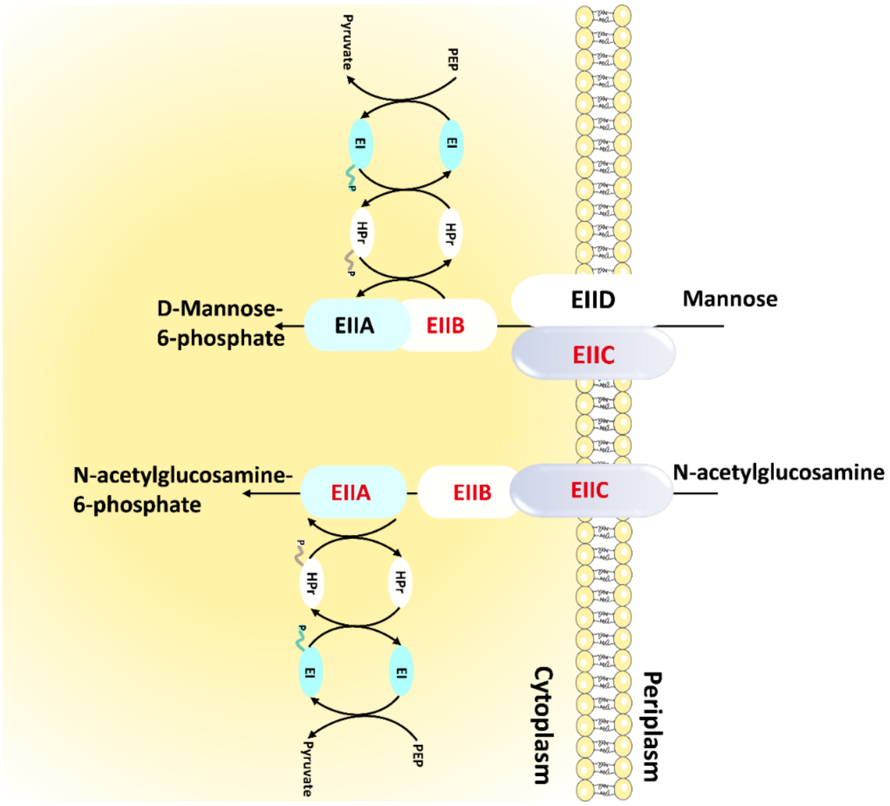

Figure S3. The specific PTS pathway in two group of strains identified by KEGG. Red represents the corresponding genes were variable in two groups.

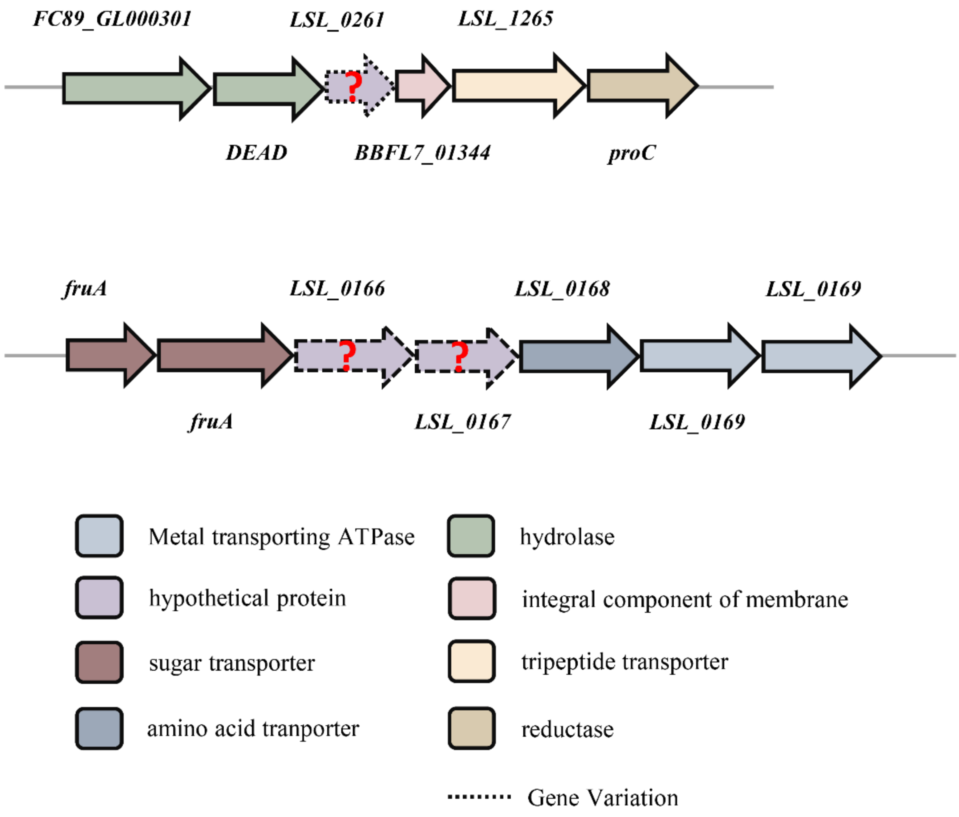

Figure S4. The upstream and downstream genes of the unknown redundant genes and their functions in *L. salivarius* strains FYNDL5M1.

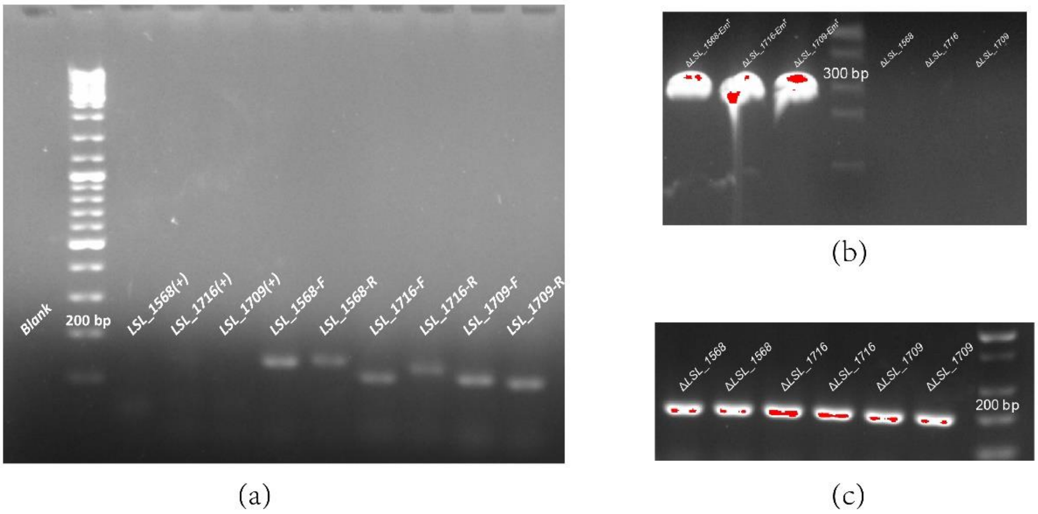

Figure S5. The DNA electrophoresis of three mutant strains. (a) Confirmation of double-crossover mutants.(+) represents the wild type strains; (b) Confirmation of the Em^r colonies. The left three bands of the DNA marker represent Em^r colonies selected via MRS plates with erythromycin. The right three bands represent the in-frame mutants deleting the Cre-expression vectors. (c) Confirmation of in-frame mutants. The PCR tests were conducted twice for each strain.
